# Blaschkolinear Acquired Inflammatory Skin Eruption (BLAISE): Case Report of a Young Man Whose Dermatosis had Features of Lichen Striatus and Blaschkitis

**DOI:** 10.7759/cureus.10785

**Published:** 2020-10-03

**Authors:** Adrija K Darsha, Philip R Cohen

**Affiliations:** 1 Medicine, University of California San Diego, La Jolla, USA; 2 Dermatology, San Diego Family Dermatology, National City, USA

**Keywords:** blaschkitis, blaschko, blaschkoid, blaschkolinear acquired inflammatory skin eruption (blaise), lichen, linear, papules, pruritus, striatus, vesicles

## Abstract

Cutaneous conditions can follow Blaschko’s lines on the skin, which are thought to reflect patterns of cell migration and clonal expansion during embryonic development of the epidermis. These diseases are hypothesized to be caused by genetic mosaicism resulting from processes such as lyonization or somatic postzygotic mutation. Lichen striatus and blaschkitis are two such acquired inflammatory skin disorders that are distinguished in the literature by age of onset, location, and histopathological features. Lichen striatus is typically observed on the extremities of children and is characterized by lichenoid papules that appear in a linear distribution along Blaschko’s lines. Microscopic examination typically shows spongiosis, as well as lichenoid and periadnexal inflammation. Blaschkitis more commonly occurs in adults and frequently involves the truncal areas, including the chest and abdomen. Microscopic examination typically shows spongiotic dermatitis. We describe a young man with a linear eruption extending from the flexor aspect of his right wrist to his central chest, which has features of both lichen striatus and blaschkitis. Both lichen striatus and blaschkitis are self-limited diseases that may resolve within months. It has been suggested that lichen striatus and blaschkitis are not separate entities, but rather the two endpoints within the spectrum of blaschkolinear acquired inflammatory skin eruption (BLAISE). The overlapping features of lichen striatus and blaschkitis in our patient demonstrate the spectrum of clinical and pathologic features in patients with BLAISE.

## Introduction

Blaschkolinear acquired inflammatory skin eruption (BLAISE) comprises a variety of dermatoses. They are characterized by inflammatory infiltrate and distribution along the lines of Blaschko. The most common of these are lichen striatus and blaschkitis, which are distinguished by age of onset, location, and histopathological features [1-3]. 

Lichen striatus is usually observed on the extremities of children. It is characterized by lichenoid papules that appear in a linear distribution along Blaschko’s lines. Microscopic examination typically shows spongiosis, as well as lichenoid and periadnexal inflammation [4-14].

Blaschkitis more commonly occurs in adults. It frequently involves the truncal areas, including the chest and abdomen. Pathologic changes demonstrate spongiotic dermatitis [15-18].

A young man developed a linear eruption extending from the flexor aspect of his right wrist to his central chest. The dermatosis had features of both lichen striatus and blaschkitis. The characteristics of lichen striatus, blaschkitis, and BLAISE are reviewed.

## Case presentation

A 20-year-old man presented with asymptomatic skin lesions of three months duration. The lesions began in the biceps area. Subsequently, they spread proximally and distally from the location, eventually extending to the central chest and the distal ventral wrist.

The patient’s past medical history was significant for hypoplastic heart syndrome, for which he had surgery at age nine years. The patient was taking ASA, lisinopril, and metoprolol for several years. He had no history of vaccinations, travel, or recent infection.

**Figure 1:**
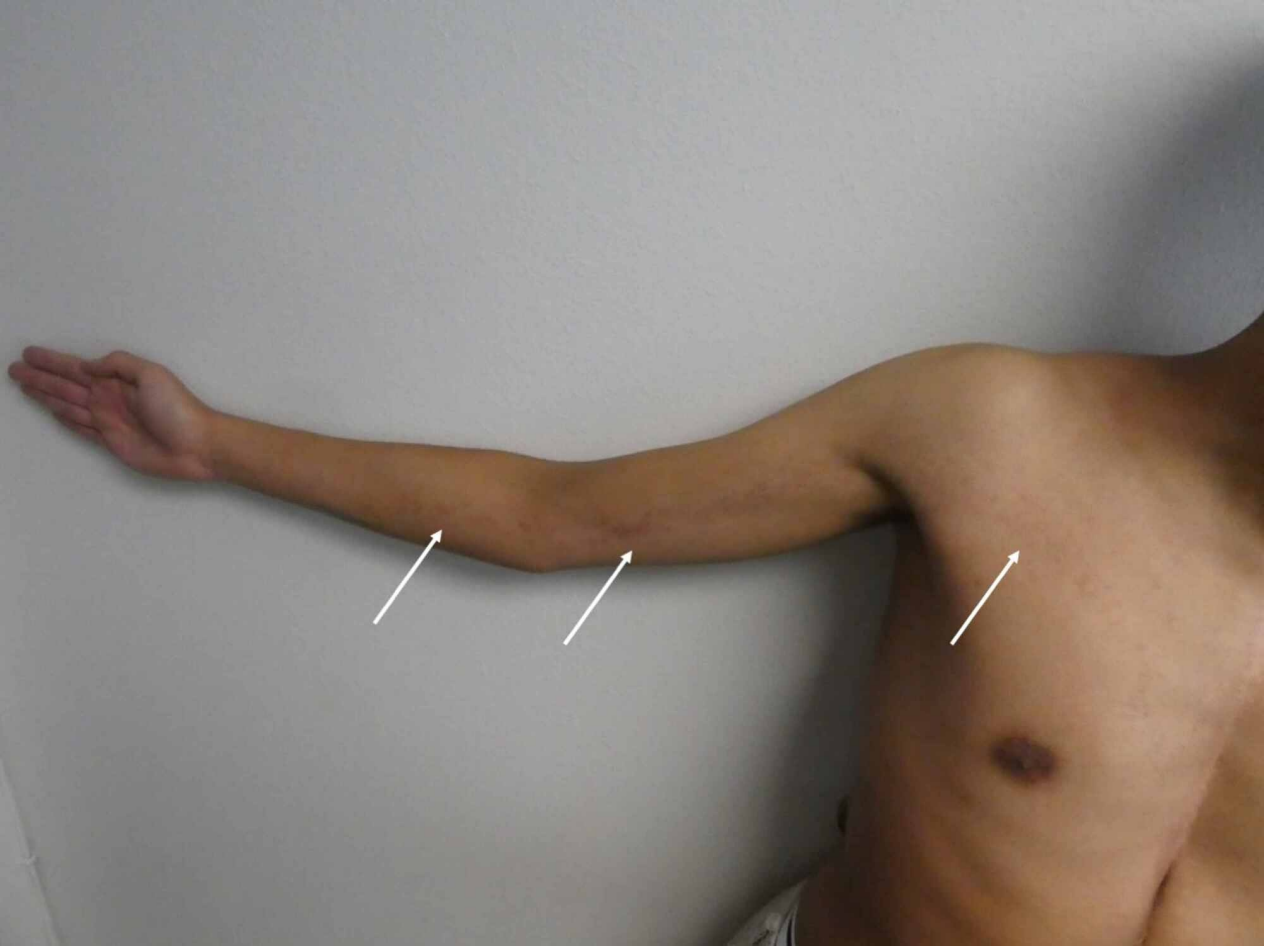
Blaschkolinear acquired inflammatory skin eruption (BLAISE) A distant view of the right upper extremity and chest of a 20-year-old man shows individual and confluent, erythematous papules (white arrows) following Blaschko’s lines on the skin.

**Figure 2:**
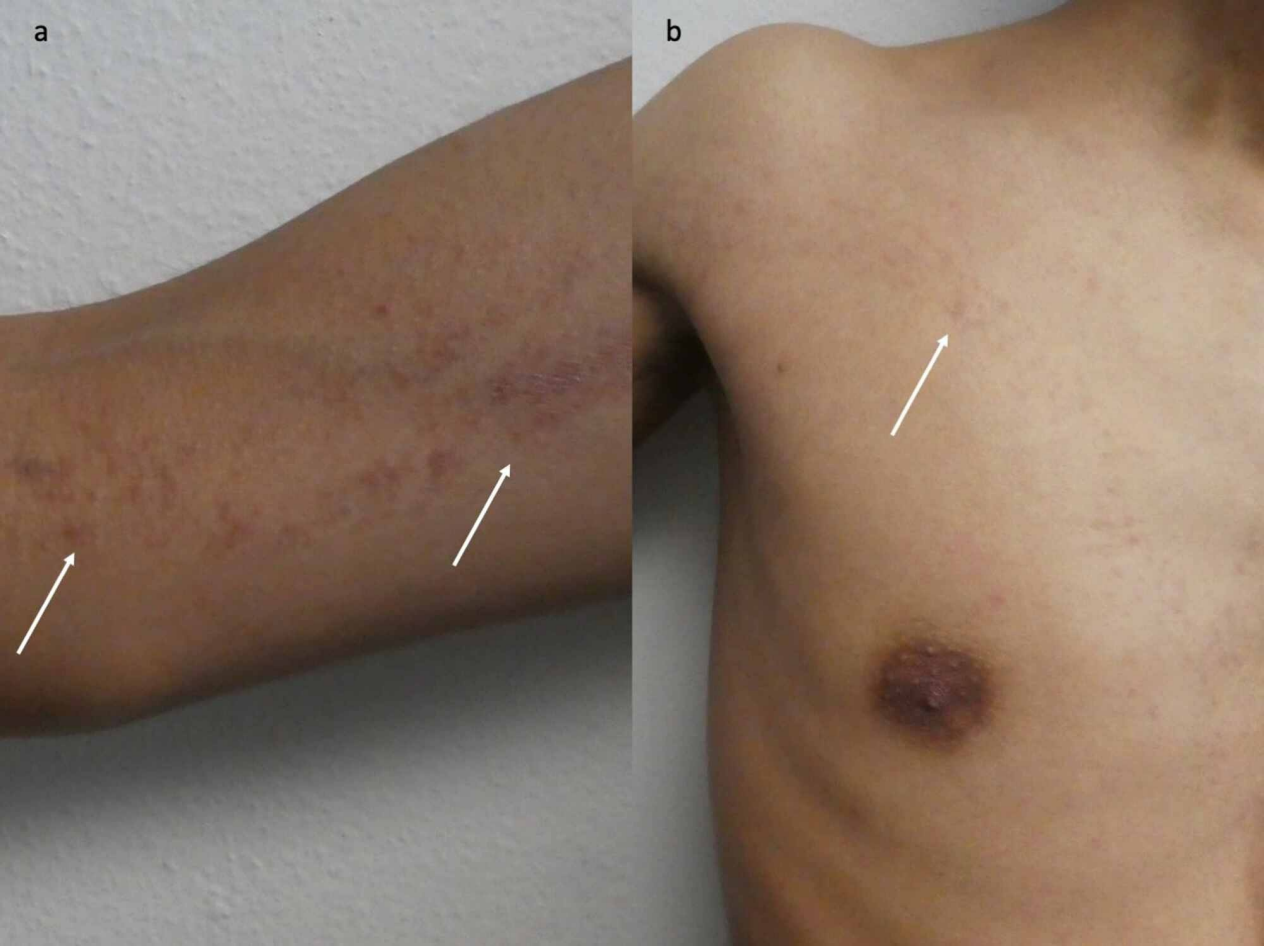
Blaschkolinear acquired inflammatory skin eruption (BLAISE) with features of both lichen striatus and blaschkitis Closer views of the right upper extremity (a) and chest (b) show erythematous papules (white arrows) following Blaschko’s lines.

Cutaneous examination revealed small individual and confluent papules in a linear pattern on the right upper extremity extending from the wrist to the mid-right central chest (Figures 1-2).

Microscopic examination of the lesions showed a superficial infiltrate of lymphocytes and histiocytes in a linear, band-like, distribution in the papillary dermis with extension along the eccrine sweat glands. Spongiosis and necrotic keratinocytes were present in the overlying epidermis. A periodic acid-Schiff stain was negative for hyphae.

**Figure 3:**
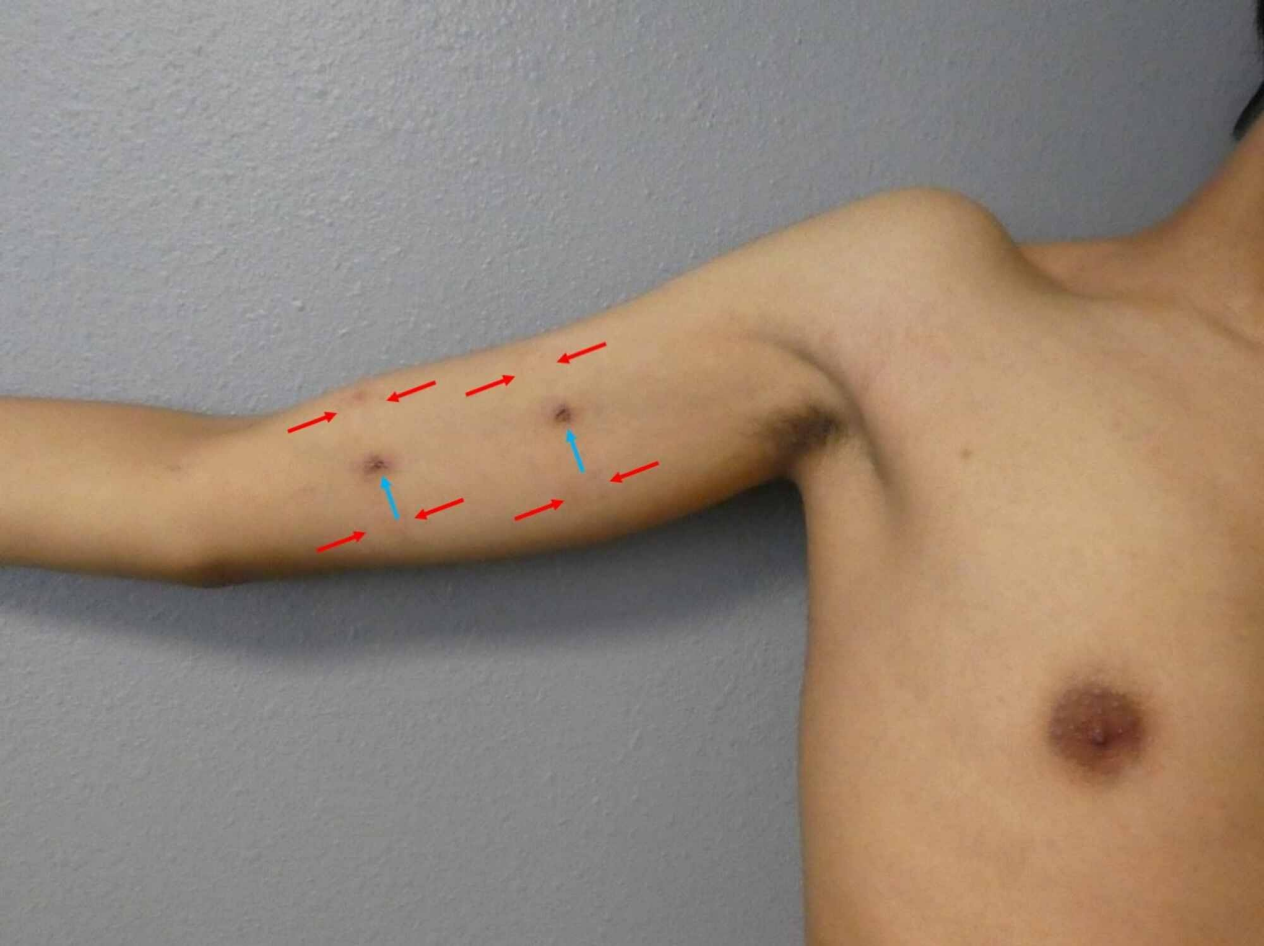
Near-complete resolution of cutaneous lesions after treatment with a topical corticosteroid cream A distant view of the patient’s right upper extremity and chest shows almost complete resolution of his blaschkolinear acquired inflammatory skin eruption (BLAISE) after two weeks of treatment with topical betamethasone dipropionate 0.05% cream. The healed biopsy sites (blue arrows) show post-inflammatory hyperpigmentation. Rectangles of erythema and hyperpigmentation (between the red arrows) demonstrated allergic contact dermatitis to the band aid adhesive.

**Figure 4:**
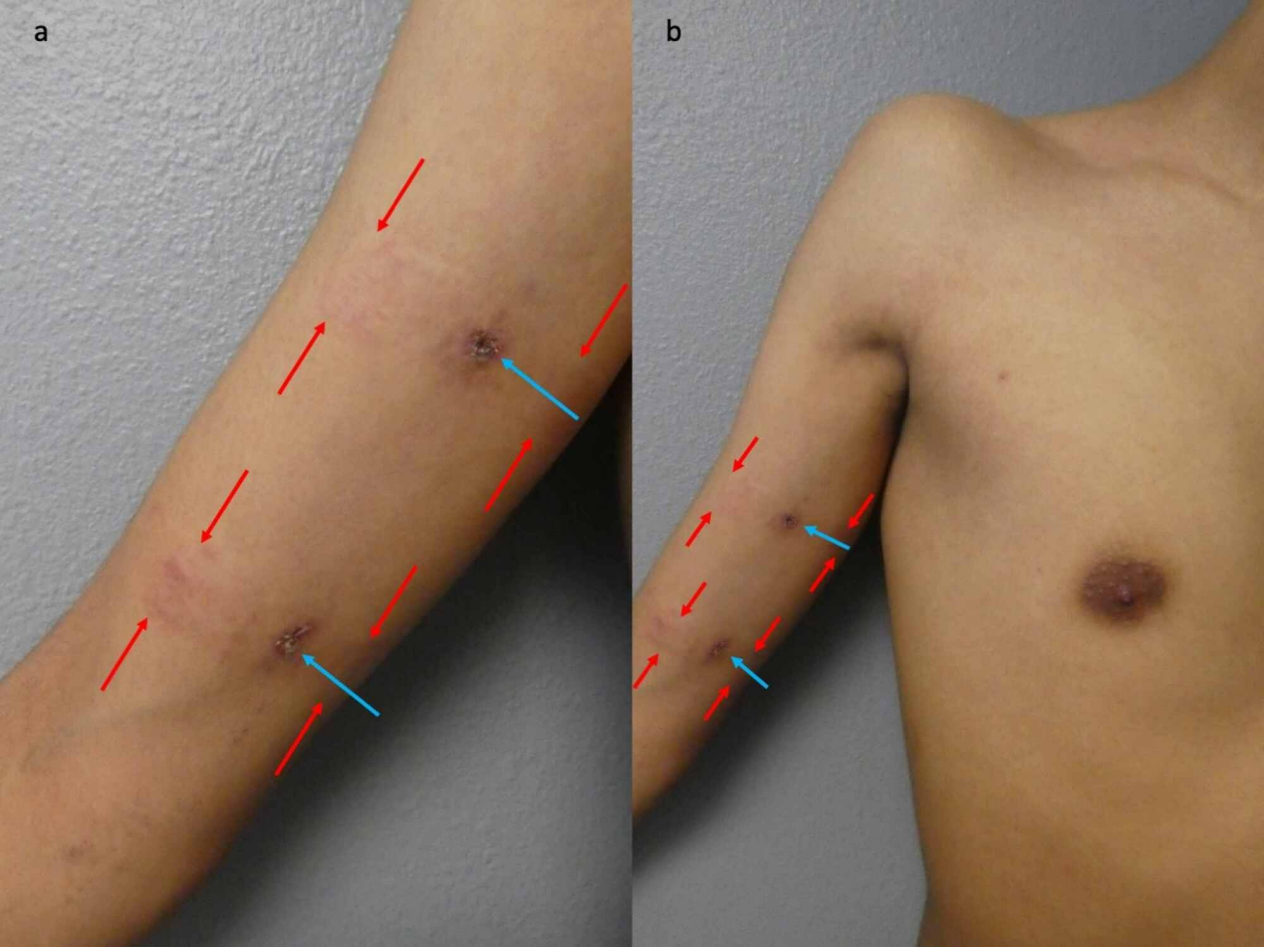
Improvement in cutaneous lesions after two weeks of topical corticosteroid treatment Closer views of the right upper extremity (a) and chest (b) show a near-complete resolution of our patient’s blaschkolinear acquired inflammatory skin eruption (BLAISE) after two weeks of treatment with high-potency betamethasone dipropionate cream. There is post-inflammatory hyperpigmentation of the two skin biopsy sites (blue arrows). Rectangles of erythema and hyperpigmentation (between the red arrows) demonstrated allergic contact dermatitis to the band aid adhesive.

Correlation of the history, clinical presentation, and pathology established a diagnosis of BLAISE with features of both lichen striatus and blaschkitis. Treatment options discussed with the patient include observation and topical corticosteroids. The patient elected to apply high potency betamethasone dipropionate 0.05% twice daily. A near-complete resolution of his skin lesions was observed after two weeks of treatment (Figures 3-4). 

## Discussion

The distribution of cutaneous conditions can morphologically present as lines on the patient’s body. These can be linear or correspond to patterns such as Blaschko’s lines, dermatomes, Langer’s lines, or Voigt’s lines, each of which has physiological etiologies. Blaschko’s lines were first described by Alfred Blaschko in 1901 and are thought to reflect patterns of cell migration and clonal expansion during the embryonic development of the epidermis [2].

The configuration of Blaschko’s lines are varied. It depends on their location. They appear in a V-shaped configuration on the back, in an S-shaped configuration on the anterior abdomen, and linearly on the extremities [2].

Congenital, nevoid, and acquired skin diseases that follow the lines of Blaschko are hypothesized to be caused by genetic mosaicism resulting from processes such as lyonization or postzygotic somatic mutation. These processes are thought to result in abnormal clones of cells that proliferate along the lines of Blaschko. They have an increased susceptibility to the development of acquired inflammatory dermatoses upon exposure to exogenous triggers such as stress, viral infections, or certain medications [2]. 

The presence of genetic mosaicism has been proven in several cases of Blaschko-linear nevoid, X-linked, and acquired inflammatory diseases. It is hypothesized that in both lichen striatus and blaschkitis, external stimuli or autoimmune responses result in an inflammatory T-cell reaction that is directed against keratinocytes. The keratinocytes have been made susceptible by genetic mosaicism that is present along the lines of Blaschko [3].

Lichen striatus is an acquired inflammatory skin disorder that predominantly affects children. The eruption follows the lines of Blaschko, involves the extremities, and is typically unilateral. Its onset is sudden, with full progression taking place over a few weeks. The etiology of lichen striatus remains unknown. Researchers suggest hypersensitivity reactions, medications, pregnancy, trauma, vaccines, and viral infections may be triggering factors. Lichen striatus has also been associated with atopy and psoriasis vulgaris (Table 1) [1,4-14,18]. 

**Table 1:** Classic clinical presentations of lichen striatus and blaschkitis

Characteristics	Lichen striatus	Blaschkitis	Our patient
Epidemiology	Children	Adults	Adult
Symptoms	Commonly asymptomatic; occasional pruritus	Pruritus may be present	Asymptomatic
Morphology	Lichenoid papules; can be erythematous	Papulovesicles; can be erythematous	Individual and confluent papules
Distribution	Extremities	Truncal areas	Right upper extremity and chest
Pathology	Dermal band-like lymphocytic infiltrate, with epidermal dyskeratosis, necrotic keratinocytes, and focal epidermal spongiosis	Epidermal spongiosis	Band-like infiltrate of lymphocytes and histiocytes, variable spongiosis, and necrotic keratinocytes
Treatment	Topical corticosteroids	Topical or systemic corticosteroids	Topical betamethasone dipropionate 0.05% cream
Response to therapy	Minimal; some patients improve with treatment	Minimal with topical corticosteroids; some patients improvement with systemic corticosteroids	Near-complete resolution of lesions after two weeks of twice-daily treatment
References	[1, 4-14]	[1, 9-14, 18]	Current report

The pathologic features of lichen striatus are variable and nonspecific. They can be characterized by a band-like infiltrate in the papillary dermis at the dermal-epidermal junction, deeper dermal lymphocytic infiltrates centered around eccrine glands and hair follicles, and/or epidermal changes such as dyskeratosis, necrotic keratinocytes, and focal spongiosis [9].

Lichen striatus typically resolves spontaneously within six to 12 months and does not recur. Topical corticosteroids may be used for the treatment of lichen striatus-associated pruritus. However, they usually do not reduce the duration of the disease or the occurrence of post-inflammatory dyspigmentation [10]. In some patients, lichen striatus has been successfully treated with other topical (pimecrolimus) or oral (acitretin, cyclosporin, or corticosteroids) agents [8,15-17].

Blaschkitis is an acquired inflammatory skin disorder that some investigators consider to be an adult variant of lichen striatus. The classic presentation of blaschkitis consists of papules and vesicles distributed along ipsilateral lines of blaschko on the truncal areas of adult patients. Histologically, it is characterized by spongiosis in the epidermis. Criteria to differentiate blaschkitis from lichen striatus in adults was established in 1999 (Table 1) [1,4-14,18].

Blaschkitis has a rapid time course, with full progression taking place over a few weeks and spontaneous resolution within two months. However, the cutaneous lesions may relapse--more than once--for months or years. Emotional stress, medications (such as certolizumab and metronidazole), trauma, and viral infections have been implicated as triggers for blaschkitis [1,11,12].

Blaschkitis has been reported to occur with features of other skin diseases such as lichen nitidus, lichen striatus, and lupus erythematosus [1,3,11]. Blaschkitis typically shows minimal response to topical corticosteroids; yet, several individuals with blaschkitis, including our patient, have experienced improvement with topical corticosteroid creams. In some patients with blaschkitis, systemic corticosteroids have achieved resolution of the cutaneous lesions [1,13].

Several researchers challenge the classification of blaschkitis and lichen striatus as distinct diseases. Indeed, some researchers have described the occurrence of blaschkitis in children and lichen striatus in adults. In addition, investigators have also demonstrated an overlap in the clinical and histologic features of these conditions [16,17,19].

Keegan et al. reported two pediatric blaschkitis patients and Fogagnolo et al. observed an asymptomatic adult with facial lichen striatus [19,20]. In addition, Raposo et al. described a 21-year-old man with erythematous papules and vesicles distributed on the left side of the abdomen and left upper limb; microscopic examination of his skin lesions showed features of both blaschkitis and lichen striatus, including hyperkeratosis, acanthosis, spongiosis, and lichenoid infiltrates in the dermis [3]. Indeed, apart from age of onset and multiple site involvement, a study by Baek et al. of 40 BLAISE patients discovered no significant differences in the clinicopathological findings of blaschkitis and lichen striatus, including disease course, relapse, spongiosis, and lichenoid infiltration [14].

Hence, several investigators consider BLAISE to be a unifying disease. It consists not only of blaschkitis and lichen striatus, but also rare, blaschkoid and linear presentations of common inflammatory dermatoses such as atopic dermatitis, graft-versus-host disease, lichen nitidus, lichen planus, lupus erythematosus, and psoriasis. Therefore, as initially proposed by Muller et al., lichen striatus and blaschkitis may not be separate entities, but rather within the spectrum of BLAISE [18].

Our patient’s clinical presentation was not uniformly pathognomonic for either blaschkitis or lichen striatus. The involvement of the chest and his adult age matched the classic clinical presentation of blaschkitis; however, the involvement of the right upper extremity was more characteristic of lichen striatus. The pathology features of his skin biopsies - an inflammatory band-like infiltrate of lymphocytes and histiocytes along with the presence of necrotic keratinocytes - was consistent with lichen striatus; yet, the presence of spongiosis is a feature that can be observed in both blaschkitis and lichen striatus. Hence, the diagnosis of BLAISE is appropriate for individuals, such as our patient, with a new dermatosis that has overlapping features of lichen striatus and blaschkitis.

## Conclusions

BLAISE includes lichen striatus and blaschkitis - self-limited acquired inflammatory dermatoses that occur along lines of Blaschko. Lichen striatus is typically found on the extremities of children and shows a dermal lichenoid infiltrate with dyskeratosis, necrotic keratinocytes, and focal spongiosis in the epidermis. In contrast, blaschkitis typically occurs on the truncal areas of adults and demonstrates epidermal spongiosis. Our patient had BLAISE with features of both lichen striatus and blaschkitis; his dermatosis promptly responded to twice daily topical application of a high-potency corticosteroid cream with a near-complete resolution after two weeks of treatment. 
